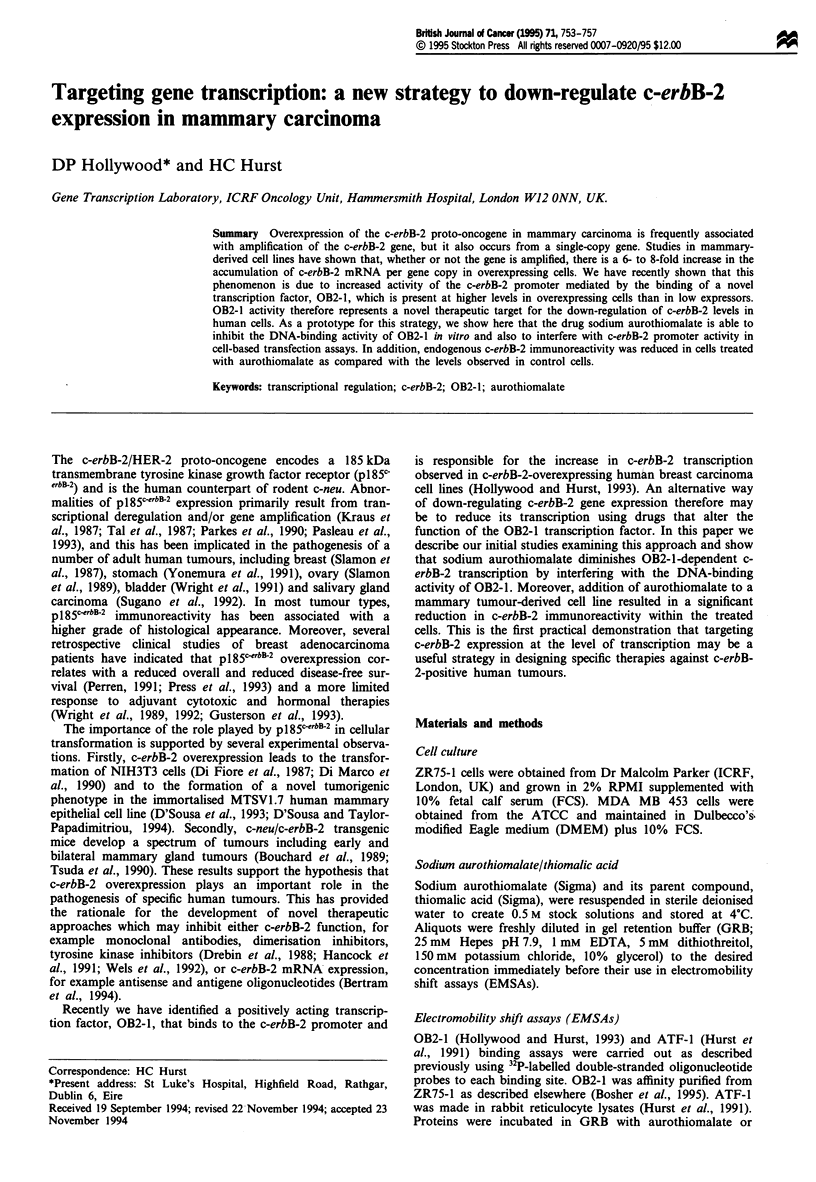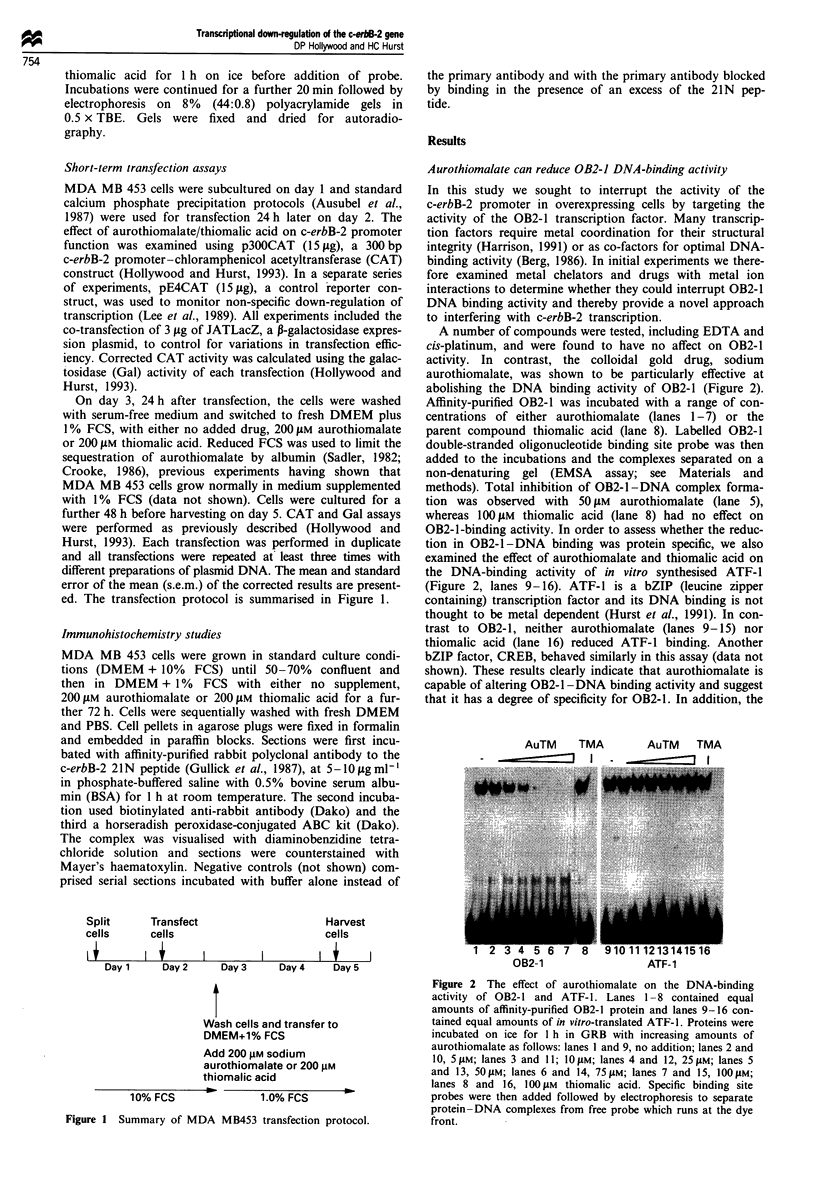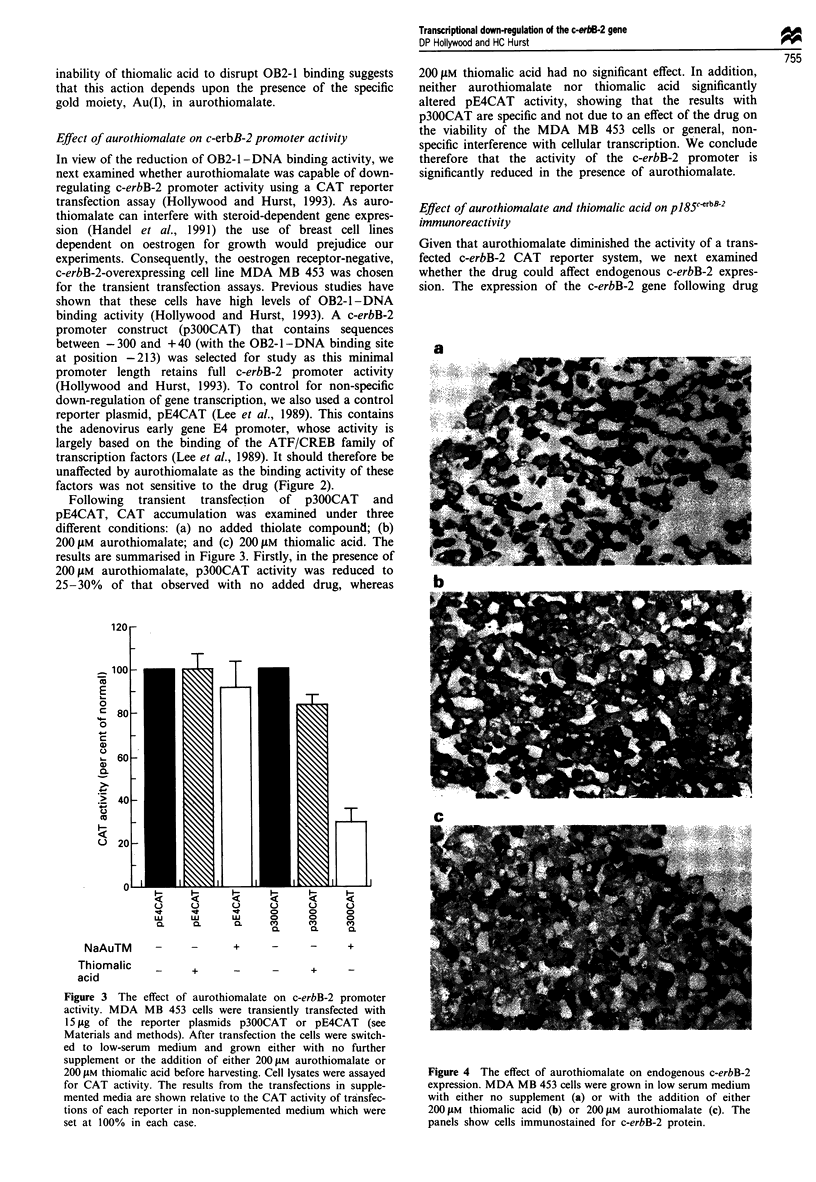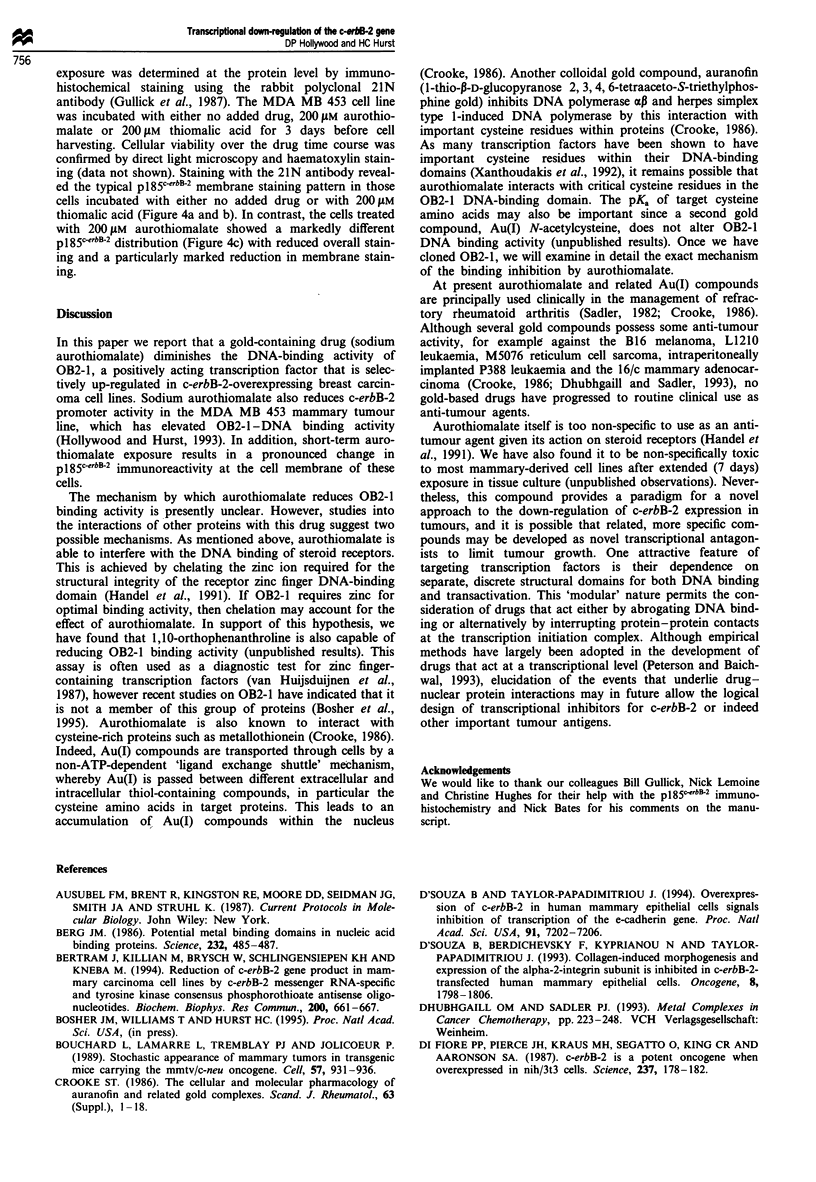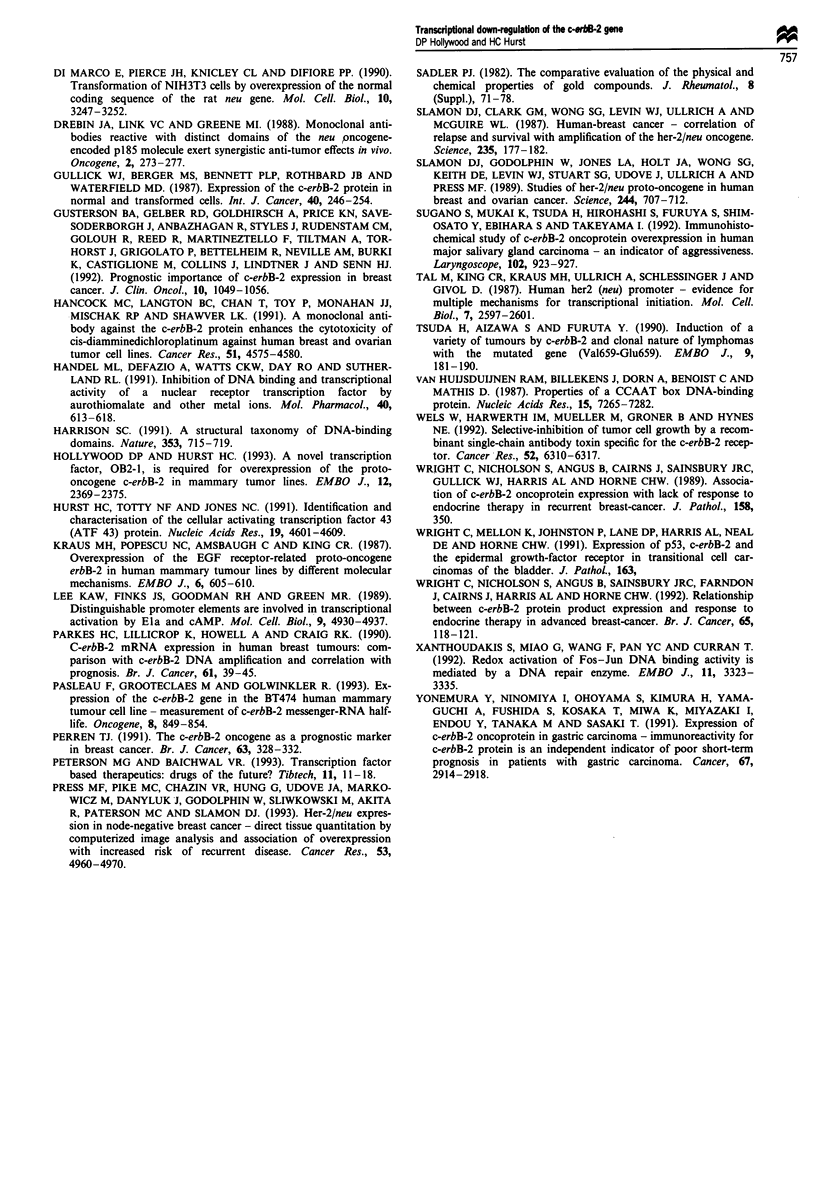# Targeting gene transcription: a new strategy to down-regulate c-erbB-2 expression in mammary carcinoma.

**DOI:** 10.1038/bjc.1995.146

**Published:** 1995-04

**Authors:** D. P. Hollywood, H. C. Hurst

**Affiliations:** Gene Transcription Laboratory, Hammersmith Hospital, London, UK.

## Abstract

**Images:**


British Journal of Cancer (1995) 71, 753-757
© 1995 Stockton Press All rights reserved 0007-0920/95 $12.00

### Targeting gene transcription: a new strategy to down-regulate c-*erb*B-2 expression in mammary carcinoma

DP Hollywood* and HC Hurst

*Gene Transcription Laboratory, ICRF Oncology Unit, Hammersmith Hospital, London W12 0NN, UK.*

**Summary** Overexpression of the c-*erb*B-2 proto-oncogene in mammary carcinoma is frequently associated with amplification of the c-*erb*B-2 gene, but it also occurs from a single-copy gene. Studies in mammary-derived cell lines have shown that, whether or not the gene is amplified, there is a 6- to 8-fold increase in the accumulation of c-*erb*B-2 mRNA per gene copy in overexpressing cells. We have recently shown that this phenomenon is due to increased activity of the c-*erb*B-2 promoter mediated by the binding of a novel transcription factor, OB2-1, which is present at higher levels in overexpressing cells than in low expressors. OB2-1 activity therefore represents a novel therapeutic target for the down-regulation of c-*erb*B-2 levels in human cells. As a prototype for this strategy, we show here that the drug sodium aurothiomalate is able to inhibit the DNA-binding activity of OB2-1 *in vitro* and also to interfere with c-*erb*B-2 promoter activity in cell-based transfection assays. In addition, endogenous c-*erb*B-2 immunoreactivity was reduced in cells treated with aurothiomalate as compared with the levels observed in control cells.

**Keywords:** transcriptional regulation; c-*erb*B-2; OB2-1; aurothiomalate

The c-*erb*B-2/HER-2 proto-oncogene encodes a 185 kDa transmembrane tyrosine kinase growth factor receptor ($p185^{c\text{-}erbB\text{-}2}$) and is the human counterpart of rodent c-*neu*. Abnormalities of $p185^{c\text{-}erbB\text{-}2}$ expression primarily result from transcriptional deregulation and/or gene amplification (Kraus *et al.*, 1987; Tal *et al.*, 1987; Parkes *et al.*, 1990; Pasleau *et al.*, 1993), and this has been implicated in the pathogenesis of a number of adult human tumours, including breast (Slamon *et al.*, 1987), stomach (Yonemura *et al.*, 1991), ovary (Slamon *et al.*, 1989), bladder (Wright *et al.*, 1991) and salivary gland carcinoma (Sugano *et al.*, 1992). In most tumour types, $p185^{c\text{-}erbB\text{-}2}$ immunoreactivity has been associated with a higher grade of histological appearance. Moreover, several retrospective clinical studies of breast adenocarcinoma patients have indicated that $p185^{c\text{-}erbB\text{-}2}$ overexpression correlates with a reduced overall and reduced disease-free survival (Perren, 1991; Press *et al.*, 1993) and a more limited response to adjuvant cytotoxic and hormonal therapies (Wright *et al.*, 1989, 1992; Gusterson *et al.*, 1993).

The importance of the role played by $p185^{c\text{-}erbB\text{-}2}$ in cellular transformation is supported by several experimental observations. Firstly, c-*erb*B-2 overexpression leads to the transformation of NIH3T3 cells (Di Fiore *et al.*, 1987; Di Marco *et al.*, 1990) and to the formation of a novel tumorigenic phenotype in the immortalised MTSV1.7 human mammary epithelial cell line (D'Sousa *et al.*, 1993; D'Sousa and Taylor-Papadimitriou, 1994). Secondly, c-*neu*/c-*erb*B-2 transgenic mice develop a spectrum of tumours including early and bilateral mammary gland tumours (Bouchard *et al.*, 1989; Tsuda *et al.*, 1990). These results support the hypothesis that c-*erb*B-2 overexpression plays an important role in the pathogenesis of specific human tumours. This has provided the rationale for the development of novel therapeutic approaches which may inhibit either c-*erb*B-2 function, for example monoclonal antibodies, dimerisation inhibitors, tyrosine kinase inhibitors (Drebin *et al.*, 1988; Hancock *et al.*, 1991; Wels *et al.*, 1992), or c-*erb*B-2 mRNA expression, for example antisense and antigene oligonucleotides (Bertram *et al.*, 1994).

Recently we have identified a positively acting transcription factor, OB2-1, that binds to the c-*erb*B-2 promoter and is responsible for the increase in c-*erb*B-2 transcription observed in c-*erb*B-2-overexpressing human breast carcinoma cell lines (Hollywood and Hurst, 1993). An alternative way of down-regulating c-*erb*B-2 gene expression therefore may be to reduce its transcription using drugs that alter the function of the OB2-1 transcription factor. In this paper we describe our initial studies examining this approach and show that sodium aurothiomalate diminishes OB2-1-dependent c-*erb*B-2 transcription by interfering with the DNA-binding activity of OB2-1. Moreover, addition of aurothiomalate to a mammary tumour-derived cell line resulted in a significant reduction in c-*erb*B-2 immunoreactivity within the treated cells. This is the first practical demonstration that targeting c-*erb*B-2 expression at the level of transcription may be a useful strategy in designing specific therapies against c-*erb*B-2-positive human tumours.

### Materials and methods

### *Cell culture*

ZR75-1 cells were obtained from Dr Malcolm Parker (ICRF, London, UK) and grown in 2% RPMI supplemented with 10% fetal calf serum (FCS). MDA MB 453 cells were obtained from the ATCC and maintained in Dulbecco's modified Eagle medium (DMEM) plus 10% FCS.

### *Sodium aurothiomalate/thiomalic acid*

Sodium aurothiomalate (Sigma) and its parent compound, thiomalic acid (Sigma), were resuspended in sterile deionised water to create 0.5 M stock solutions and stored at 4°C. Aliquots were freshly diluted in gel retention buffer (GRB; 25 mM Hepes pH 7.9, 1 mM EDTA, 5 mM dithiothreitol, 150 mM potassium chloride, 10% glycerol) to the desired concentration immediately before their use in electromobility shift assays (EMSAs).

### *Electromobility shift assays (EMSAs)*

OB2-1 (Hollywood and Hurst, 1993) and ATF-1 (Hurst *et al.*, 1991) binding assays were carried out as described previously using $^{32}P$-labelled double-stranded oligonucleotide probes to each binding site. OB2-1 was affinity purified from ZR75-1 as described elsewhere (Bosher *et al.*, 1995). ATF-1 was made in rabbit reticulocyte lysates (Hurst *et al.*, 1991). Proteins were incubated in GRB with aurothiomalate or

Correspondence: HC Hurst
*Present address: St Luke's Hospital, Highfield Road, Rathgar, Dublin 6, Eire
Received 19 September 1994; revised 22 November 1994; accepted 23 November 1994

thiomalic acid for 1 h on ice before addition of probe. Incubations were continued for a further 20 min followed by electrophoresis on 8% (44:0.8) polyacrylamide gels in 0.5 × TBE. Gels were fixed and dried for autoradiography.

*Short-term transfection assays*

MDA MB 453 cells were subcultured on day 1 and standard calcium phosphate precipitation protocols (Ausubel *et al.*, 1987) were used for transfection 24 h later on day 2. The effect of aurothiomalate/thiomalic acid on c-*erb*B-2 promoter function was examined using p300CAT (15 μg), a 300 bp c-*erb*B-2 promoter–chloramphenicol acetyltransferase (CAT) construct (Hollywood and Hurst, 1993). In a separate series of experiments, pE4CAT (15 μg), a control reporter construct, was used to monitor non-specific down-regulation of transcription (Lee *et al.*, 1989). All experiments included the co-transfection of 3 μg of JATLacZ, a β-galactosidase expression plasmid, to control for variations in transfection efficiency. Corrected CAT activity was calculated using the galactosidase (Gal) activity of each transfection (Hollywood and Hurst, 1993).

On day 3, 24 h after transfection, the cells were washed with serum-free medium and switched to fresh DMEM plus 1% FCS, with either no added drug, 200 μM aurothiomalate or 200 μM thiomalic acid. Reduced FCS was used to limit the sequestration of aurothiomalate by albumin (Sadler, 1982; Crooke, 1986), previous experiments having shown that MDA MB 453 cells grow normally in medium supplemented with 1% FCS (data not shown). Cells were cultured for a further 48 h before harvesting on day 5. CAT and Gal assays were performed as previously described (Hollywood and Hurst, 1993). Each transfection was performed in duplicate and all transfections were repeated at least three times with different preparations of plasmid DNA. The mean and standard error of the mean (s.e.m.) of the corrected results are presented. The transfection protocol is summarised in Figure 1.

*Immunohistochemistry studies*

MDA MB 453 cells were grown in standard culture conditions (DMEM + 10% FCS) until 50–70% confluent and then in DMEM + 1% FCS with either no supplement, 200 μM aurothiomalate or 200 μM thiomalic acid for a further 72 h. Cells were sequentially washed with fresh DMEM and PBS. Cell pellets in agarose plugs were fixed in formalin and embedded in paraffin blocks. Sections were first incubated with affinity-purified rabbit polyclonal antibody to the c-*erb*B-2 21N peptide (Gullick *et al.*, 1987), at 5–10 μg $ml^{-1}$ in phosphate-buffered saline with 0.5% bovine serum albumin (BSA) for 1 h at room temperature. The second incubation used biotinylated anti-rabbit antibody (Dako) and the third a horseradish peroxidase-conjugated ABC kit (Dako). The complex was visualised with diaminobenzidine tetrachloride solution and sections were counterstained with Mayer's haematoxylin. Negative controls (not shown) comprised serial sections incubated with buffer alone instead of the primary antibody and with the primary antibody blocked by binding in the presence of an excess of the 21N peptide.

**Figure 1** Summary of MDA MB453 transfection protocol.

### Results

*Aurothiomalate can reduce OB2-1 DNA-binding activity*

In this study we sought to interrupt the activity of the c-*erb*B-2 promoter in overexpressing cells by targeting the activity of the OB2-1 transcription factor. Many transcription factors require metal coordination for their structural integrity (Harrison, 1991) or as co-factors for optimal DNA-binding activity (Berg, 1986). In initial experiments we therefore examined metal chelators and drugs with metal ion interactions to determine whether they could interrupt OB2-1 DNA binding activity and thereby provide a novel approach to interfering with c-*erb*B-2 transcription.

A number of compounds were tested, including EDTA and *cis*-platinum, and were found to have no affect on OB2-1 activity. In contrast, the colloidal gold drug, sodium aurothiomalate, was shown to be particularly effective at abolishing the DNA binding activity of OB2-1 (Figure 2). Affinity-purified OB2-1 was incubated with a range of concentrations of either aurothiomalate (lanes 1–7) or the parent compound thiomalic acid (lane 8). Labelled OB2-1 double-stranded oligonucleotide binding site probe was then added to the incubations and the complexes separated on a non-denaturing gel (EMSA assay; see Materials and methods). Total inhibition of OB2-1–DNA complex formation was observed with 50 μM aurothiomalate (lane 5), whereas 100 μM thiomalic acid (lane 8) had no effect on OB2-1-binding activity. In order to assess whether the reduction in OB2-1–DNA binding was protein specific, we also examined the effect of aurothiomalate and thiomalic acid on the DNA-binding activity of *in vitro* synthesised ATF-1 (Figure 2, lanes 9–16). ATF-1 is a bZIP (leucine zipper containing) transcription factor and its DNA binding is not thought to be metal dependent (Hurst *et al.*, 1991). In contrast to OB2-1, neither aurothiomalate (lanes 9–15) nor thiomalic acid (lane 16) reduced ATF-1 binding. Another bZIP factor, CREB, behaved similarly in this assay (data not shown). These results clearly indicate that aurothiomalate is capable of altering OB2-1–DNA binding activity and suggest that it has a degree of specificity for OB2-1. In addition, the

**Figure 2** The effect of aurothiomalate on the DNA-binding activity of OB2-1 and ATF-1. Lanes 1–8 contained equal amounts of affinity-purified OB2-1 protein and lanes 9–16 contained equal amounts of *in vitro*-translated ATF-1. Proteins were incubated on ice for 1 h in GRB with increasing amounts of aurothiomalate as follows: lanes 1 and 9, no addition; lanes 2 and 10, 5 μM; lanes 3 and 11; 10 μM; lanes 4 and 12, 25 μM; lanes 5 and 13, 50 μM; lanes 6 and 14, 75 μM; lanes 7 and 15, 100 μM; lanes 8 and 16, 100 μM thiomalic acid. Specific binding site probes were then added followed by electrophoresis to separate protein–DNA complexes from free probe which runs at the dye front.

inability of thiomalic acid to disrupt OB2-1 binding suggests that this action depends upon the presence of the specific gold moiety, Au(I), in aurothiomalate.

*Effect of aurothiomalate on c-*erb*B-2 promoter activity*

In view of the reduction of OB2-1–DNA binding activity, we next examined whether aurothiomalate was capable of down-regulating c-*erb*B-2 promoter activity using a CAT reporter transfection assay (Hollywood and Hurst, 1993). As aurothiomalate can interfere with steroid-dependent gene expression (Handel *et al.*, 1991) the use of breast cell lines dependent on oestrogen for growth would prejudice our experiments. Consequently, the oestrogen receptor-negative, c-*erb*B-2-overexpressing cell line MDA MB 453 was chosen for the transient transfection assays. Previous studies have shown that these cells have high levels of OB2-1–DNA binding activity (Hollywood and Hurst, 1993). A c-*erb*B-2 promoter construct (p300CAT) that contains sequences between −300 and +40 (with the OB2-1–DNA binding site at position −213) was selected for study as this minimal promoter length retains full c-*erb*B-2 promoter activity (Hollywood and Hurst, 1993). To control for non-specific down-regulation of gene transcription, we also used a control reporter plasmid, pE4CAT (Lee *et al.*, 1989). This contains the adenovirus early gene E4 promoter, whose activity is largely based on the binding of the ATF/CREB family of transcription factors (Lee *et al.*, 1989). It should therefore be unaffected by aurothiomalate as the binding activity of these factors was not sensitive to the drug (Figure 2).

Following transient transfection of p300CAT and pE4CAT, CAT accumulation was examined under three different conditions: (a) no added thiolate compound; (b) 200 μM aurothiomalate; and (c) 200 μM thiomalic acid. The results are summarised in Figure 3. Firstly, in the presence of 200 μM aurothiomalate, p300CAT activity was reduced to 25–30% of that observed with no added drug, whereas 200 μM thiomalic acid had no significant effect. In addition, neither aurothiomalate nor thiomalic acid significantly altered pE4CAT activity, showing that the results with p300CAT are specific and not due to an effect of the drug on the viability of the MDA MB 453 cells or general, non-specific interference with cellular transcription. We conclude therefore that the activity of the c-*erb*B-2 promoter is significantly reduced in the presence of aurothiomalate.

*Effect of aurothiomalate and thiomalic acid on p185*$^{c\text{-}erbB\text{-}2}$ *immunoreactivity*

Given that aurothiomalate diminished the activity of a transfected c-*erb*B-2 CAT reporter system, we next examined whether the drug could affect endogenous c-*erb*B-2 expression. The expression of the c-*erb*B-2 gene following drug

**Figure 3** The effect of aurothiomalate on c-*erb*B-2 promoter activity. MDA MB 453 cells were transiently transfected with 15 μg of the reporter plasmids p300CAT or pE4CAT (see Materials and methods). After transfection the cells were switched to low-serum medium and grown either with no further supplement or the addition of either 200 μM aurothiomalate or 200 μM thiomalic acid before harvesting. Cell lysates were assayed for CAT activity. The results from the transfections in supplemented media are shown relative to the CAT activity of transfections of each reporter in non-supplemented medium which were set at 100% in each case.

**Figure 4** The effect of aurothiomalate on endogenous c-*erb*B-2 expression. MDA MB 453 cells were grown in low serum medium with either no supplement (**a**) or with the addition of either 200 μM thiomalic acid (**b**) or 200 μM aurothiomalate (**c**). The panels show cells immunostained for c-*erb*B-2 protein.

exposure was determined at the protein level by immunohistochemical staining using the rabbit polyclonal 21N antibody (Gullick *et al.*, 1987). The MDA MB 453 cell line was incubated with either no added drug, 200 $\mu$M aurothiomalate or 200 $\mu$M thiomalic acid for 3 days before cell harvesting. Cellular viability over the drug time course was confirmed by direct light microscopy and haematoxylin staining (data not shown). Staining with the 21N antibody revealed the typical $p185^{c\text{-}erbB\text{-}2}$ membrane staining pattern in those cells incubated with either no added drug or with 200 $\mu$M thiomalic acid (Figure 4a and b). In contrast, the cells treated with 200 $\mu$M aurothiomalate showed a markedly different $p185^{c\text{-}erbB\text{-}2}$ distribution (Figure 4c) with reduced overall staining and a particularly marked reduction in membrane staining.

### Discussion

In this paper we report that a gold-containing drug (sodium aurothiomalate) diminishes the DNA-binding activity of OB2-1, a positively acting transcription factor that is selectively up-regulated in c-*erb*B-2-overexpressing breast carcinoma cell lines. Sodium aurothiomalate also reduces c-*erb*B-2 promoter activity in the MDA MB 453 mammary tumour line, which has elevated OB2-1-DNA binding activity (Hollywood and Hurst, 1993). In addition, short-term aurothiomalate exposure results in a pronounced change in $p185^{c\text{-}erbB\text{-}2}$ immunoreactivity at the cell membrane of these cells.

The mechanism by which aurothiomalate reduces OB2-1 binding activity is presently unclear. However, studies into the interactions of other proteins with this drug suggest two possible mechanisms. As mentioned above, aurothiomalate is able to interfere with the DNA binding of steroid receptors. This is achieved by chelating the zinc ion required for the structural integrity of the receptor zinc finger DNA-binding domain (Handel *et al.*, 1991). If OB2-1 requires zinc for optimal binding activity, then chelation may account for the effect of aurothiomalate. In support of this hypothesis, we have found that 1,10-orthophenanthroline is also capable of reducing OB2-1 binding activity (unpublished results). This assay is often used as a diagnostic test for zinc finger-containing transcription factors (van Huijsduijnen *et al.*, 1987), however recent studies on OB2-1 have indicated that it is not a member of this group of proteins (Bosher *et al.*, 1995). Aurothiomalate is also known to interact with cysteine-rich proteins such as metallothionein (Crooke, 1986). Indeed, Au(I) compounds are transported through cells by a non-ATP-dependent 'ligand exchange shuttle' mechanism, whereby Au(I) is passed between different extracellular and intracellular thiol-containing compounds, in particular the cysteine amino acids in target proteins. This leads to an accumulation of Au(I) compounds within the nucleus (Crooke, 1986). Another colloidal gold compound, auranofin (1-thio-$\beta$-D-glucopyranose 2, 3, 4, 6-tetraaceto-*S*-triethylphosphine gold) inhibits DNA polymerase $\alpha\beta$ and herpes simplex type 1-induced DNA polymerase by this interaction with important cysteine residues within proteins (Crooke, 1986). As many transcription factors have been shown to have important cysteine residues within their DNA-binding domains (Xanthoudakis *et al.*, 1992), it remains possible that aurothiomalate interacts with critical cysteine residues in the OB2-1 DNA-binding domain. The $pK_a$ of target cysteine amino acids may also be important since a second gold compound, Au(I) *N*-acetylcysteine, does not alter OB2-1 DNA binding activity (unpublished results). Once we have cloned OB2-1, we will examine in detail the exact mechanism of the binding inhibition by aurothiomalate.

At present aurothiomalate and related Au(I) compounds are principally used clinically in the management of refractory rheumatoid arthritis (Sadler, 1982; Crooke, 1986). Although several gold compounds possess some anti-tumour activity, for example against the B16 melanoma, L1210 leukaemia, M5076 reticulum cell sarcoma, intraperitoneally implanted P388 leukaemia and the 16/c mammary adenocarcinoma (Crooke, 1986; Dhubhgaill and Sadler, 1993), no gold-based drugs have progressed to routine clinical use as anti-tumour agents.

Aurothiomalate itself is too non-specific to use as an anti-tumour agent given its action on steroid receptors (Handel *et al.*, 1991). We have also found it to be non-specifically toxic to most mammary-derived cell lines after extended (7 days) exposure in tissue culture (unpublished observations). Nevertheless, this compound provides a paradigm for a novel approach to the down-regulation of c-*erb*B-2 expression in tumours, and it is possible that related, more specific compounds may be developed as novel transcriptional antagonists to limit tumour growth. One attractive feature of targeting transcription factors is their dependence on separate, discrete structural domains for both DNA binding and transactivation. This 'modular' nature permits the consideration of drugs that act either by abrogating DNA binding or alternatively by interrupting protein-protein contacts at the transcription initiation complex. Although empirical methods have largely been adopted in the development of drugs that act at a transcriptional level (Peterson and Baichwal, 1993), elucidation of the events that underlie drug-nuclear protein interactions may in future allow the logical design of transcriptional inhibitors for c-*erb*B-2 or indeed other important tumour antigens.

**Acknowledgements**
We would like to thank our colleagues Bill Gullick, Nick Lemoine and Christine Hughes for their help with the $p185^{c\text{-}erbB\text{-}2}$ immunohistochemistry and Nick Bates for his comments on the manuscript.

### References

AUSUBEL FM, BRENT R, KINGSTON RE, MOORE DD, SEIDMAN JG, SMITH JA AND STRUHL K. (1987). *Current Protocols in Molecular Biology*. John Wiley: New York.

BERG JM. (1986). Potential metal binding domains in nucleic acid binding proteins. *Science*, **232**, 485-487.

BERTRAM J, KILLIAN M, BRYSCH W, SCHLINGENSIEPEN KH AND KNEBA M. (1994). Reduction of c-*erb*B-2 gene product in mammary carcinoma cell lines by c-*erb*B-2 messenger RNA-specific and tyrosine kinase consensus phosphorothioate antisense oligonucleotides. *Biochem. Biophys. Res Commun.*, **200**, 661-667.

BOSHER JM, WILLIAMS T AND HURST HC. (1995). *Proc. Natl Acad. Sci. USA*, (in press).

BOUCHARD L, LAMARRE L, TREMBLAY PJ AND JOLICOEUR P. (1989). Stochastic appearance of mammary tumors in transgenic mice carrying the mmtv/c-*neu* oncogene. *Cell*, **57**, 931-936.

CROOKE ST. (1986). The cellular and molecular pharmacology of auranofin and related gold complexes. *Scand. J. Rheumatol.*, **63** (Suppl.), 1-18.

D'SOUZA B AND TAYLOR-PAPADIMITRIOU J. (1994). Overexpression of c-*erb*B-2 in human mammary epithelial cells signals inhibition of transcription of the e-cadherin gene. *Proc. Natl Acad. Sci. USA*, **91**, 7202-7206.

D'SOUZA B, BERDICHEVSKY F, KYPRIANOU N AND TAYLOR-PAPADIMITRIOU J. (1993). Collagen-induced morphogenesis and expression of the alpha-2-integrin subunit is inhibited in c-*erb*B-2-transfected human mammary epithelial cells. *Oncogene*, **8**, 1798-1806.

DHUBHGAILL OM AND SADLER PJ. (1993). *Metal Complexes in Cancer Chemotherapy*, pp. 223-248. VCH Verlagsgesellschaft: Weinheim.

DI FIORE PP, PIERCE JH, KRAUS MH, SEGATTO O, KING CR AND AARONSON SA. (1987). c-*erb*B-2 is a potent oncogene when overexpressed in nih/3t3 cells. *Science*, **237**, 178-182.

DI MARCO E, PIERCE JH, KNICLEY CL AND DIFIORE PP. (1990). Transformation of NIH3T3 cells by overexpression of the normal coding sequence of the rat *neu* gene. *Mol. Cell. Biol.*, **10**, 3247–3252.

DREBIN JA, LINK VC AND GREENE MI. (1988). Monoclonal antibodies reactive with distinct domains of the *neu* oncogene-encoded p185 molecule exert synergistic anti-tumor effects *in vivo*. *Oncogene*, **2**, 273–277.

GULLICK WJ, BERGER MS, BENNETT PLP, ROTHBARD JB AND WATERFIELD MD. (1987). Expression of the c-*erb*B-2 protein in normal and transformed cells. *Int. J. Cancer*, **40**, 246–254.

GUSTERSON BA, GELBER RD, GOLDHIRSCH A, PRICE KN, SAVE-SODERBORGH J, ANBAZHAGAN R, STYLES J, RUDENSTAM CM, GOLOUH R, REED R, MARTINEZTELLO F, TILTMAN A, TORHORST J, GRIGOLATO P, BETTELHEIM R, NEVILLE AM, BURKI K, CASTIGLIONE M, COLLINS J, LINDTNER J AND SENN HJ. (1992). Prognostic importance of c-*erb*B-2 expression in breast cancer. *J. Clin. Oncol.*, **10**, 1049–1056.

HANCOCK MC, LANGTON BC, CHAN T, TOY P, MONAHAN JJ, MISCHAK RP AND SHAWVER LK. (1991). A monoclonal antibody against the c-*erb*B-2 protein enhances the cytotoxicity of cis-diamminedichloroplatinum against human breast and ovarian tumor cell lines. *Cancer Res.*, **51**, 4575–4580.

HANDEL ML, DEFAZIO A, WATTS CKW, DAY RO AND SUTHERLAND RL. (1991). Inhibition of DNA binding and transcriptional activity of a nuclear receptor transcription factor by aurothiomalate and other metal ions. *Mol. Pharmacol.*, **40**, 613–618.

HARRISON SC. (1991). A structural taxonomy of DNA-binding domains. *Nature*, **353**, 715–719.

HOLLYWOOD DP AND HURST HC. (1993). A novel transcription factor, OB2-1, is required for overexpression of the proto-oncogene c-*erb*B-2 in mammary tumor lines. *EMBO J.*, **12**, 2369–2375.

HURST HC, TOTTY NF AND JONES NC. (1991). Identification and characterisation of the cellular activating transcription factor 43 (ATF 43) protein. *Nucleic Acids Res.*, **19**, 4601–4609.

KRAUS MH, POPESCU NC, AMSBAUGH C AND KING CR. (1987). Overexpression of the EGF receptor-related proto-oncogene *erb*B-2 in human mammary tumour lines by different molecular mechanisms. *EMBO J.*, **6**, 605–610.

LEE KAW, FINKS JS, GOODMAN RH AND GREEN MR. (1989). Distinguishable promoter elements are involved in transcriptional activation by Ela and cAMP. *Mol. Cell. Biol.*, **9**, 4930–4937.

PARKES HC, LILLICROP K, HOWELL A AND CRAIG RK. (1990). C-*erb*B-2 mRNA expression in human breast tumours: comparison with c-*erb*B-2 DNA amplification and correlation with prognosis. *Br. J. Cancer*, **61**, 39–45.

PASLEAU F, GROOTECLAES M AND GOLWINKLER R. (1993). Expression of the c-*erb*B-2 gene in the BT474 human mammary tumour cell line – measurement of c-*erb*B-2 messenger-RNA half-life. *Oncogene*, **8**, 849–854.

PERREN TJ. (1991). The c-*erb*B-2 oncogene as a prognostic marker in breast cancer. *Br. J. Cancer*, **63**, 328–332.

PETERSON MG AND BAICHWAL VR. (1993). Transcription factor based therapeutics: drugs of the future? *Tibtech*, **11**, 11–18.

PRESS MF, PIKE MC, CHAZIN VR, HUNG G, UDOVE JA, MARKOWICZ M, DANYLUK J, GODOLPHIN W, SLIWKOWSKI M, AKITA R, PATERSON MC AND SLAMON DJ. (1993). Her-2/*neu* expression in node-negative breast cancer – direct tissue quantitation by computerized image analysis and association of overexpression with increased risk of recurrent disease. *Cancer Res.*, **53**, 4960–4970.

SADLER PJ. (1982). The comparative evaluation of the physical and chemical properties of gold compounds. *J. Rheumatol.*, **8** (Suppl.), 71–78.

SLAMON DJ, CLARK GM, WONG SG, LEVIN WJ, ULLRICH A AND MCGUIRE WL. (1987). Human-breast cancer – correlation of relapse and survival with amplification of the her-2/*neu* oncogene. *Science*, **235**, 177–182.

SLAMON DJ, GODOLPHIN W, JONES LA, HOLT JA, WONG SG, KEITH DE, LEVIN WJ, STUART SG, UDOVE J, ULLRICH A AND PRESS MF. (1989). Studies of her-2/*neu* proto-oncogene in human breast and ovarian cancer. *Science*, **244**, 707–712.

SUGANO S, MUKAI K, TSUDA H, HIROHASHI S, FURUYA S, SHIMOSATO Y, EBIHARA S AND TAKEYAMA I. (1992). Immunohistochemical study of c-*erb*B-2 oncoprotein overexpression in human major salivary gland carcinoma – an indicator of aggressiveness. *Laryngoscope*, **102**, 923–927.

TAL M, KING CR, KRAUS MH, ULLRICH A, SCHLESSINGER J AND GIVOL D. (1987). Human her2 (*neu*) promoter – evidence for multiple mechanisms for transcriptional initiation. *Mol. Cell. Biol.*, **7**, 2597–2601.

TSUDA H, AIZAWA S AND FURUTA Y. (1990). Induction of a variety of tumours by c-*erb*B-2 and clonal nature of lymphomas with the mutated gene (Val659-Glu659). *EMBO J.*, **9**, 181–190.

VAN HUIJSDUIJNEN RAM, BILLEKENS J, DORN A, BENOIST C AND MATHIS D. (1987). Properties of a CCAAT box DNA-binding protein. *Nucleic Acids Res.*, **15**, 7265–7282.

WELS W, HARWERTH IM, MUELLER M, GRONER B AND HYNES NE. (1992). Selective-inhibition of tumor cell growth by a recombinant single-chain antibody toxin specific for the c-*erb*B-2 receptor. *Cancer Res.*, **52**, 6310–6317.

WRIGHT C, NICHOLSON S, ANGUS B, CAIRNS J, SAINSBURY JRC, GULLICK WJ, HARRIS AL AND HORNE CHW. (1989). Association of c-*erb*B-2 oncoprotein expression with lack of response to endocrine therapy in recurrent breast-cancer. *J. Pathol.*, **158**, 350.

WRIGHT C, MELLON K, JOHNSTON P, LANE DP, HARRIS AL, NEAL DE AND HORNE CHW. (1991). Expression of p53, c-*erb*B-2 and the epidermal growth-factor receptor in transitional cell carcinomas of the bladder. *J. Pathol.*, **163**,

WRIGHT C, NICHOLSON S, ANGUS B, SAINSBURY JRC, FARNDON J, CAIRNS J, HARRIS AL AND HORNE CHW. (1992). Relationship between c-*erb*B-2 protein product expression and response to endocrine therapy in advanced breast-cancer. *Br. J. Cancer*, **65**, 118–121.

XANTHOUDAKIS S, MIAO G, WANG F, PAN YC AND CURRAN T. (1992). Redox activation of Fos–Jun DNA binding activity is mediated by a DNA repair enzyme. *EMBO J.*, **11**, 3323–3335.

YONEMURA Y, NINOMIYA I, OHOYAMA S, KIMURA H, YAMAGUCHI A, FUSHIDA S, KOSAKA T, MIWA K, MIYAZAKI I, ENDOU Y, TANAKA M AND SASAKI T. (1991). Expression of c-*erb*B-2 oncoprotein in gastric carcinoma – immunoreactivity for c-*erb*B-2 protein is an independent indicator of poor short-term prognosis in patients with gastric carcinoma. *Cancer*, **67**, 2914–2918.